# Editorial: Medicinal chemistry of active pharmaceutical ingredients of drug products

**DOI:** 10.3389/fchem.2024.1409042

**Published:** 2024-04-17

**Authors:** Mohd Imran, Qasim M. Alhadidi, Shah Alam Khan, Hamdy Khamees Thabet, Ozair Alam, Faiyaz Shakeel, Ziyaur Rahman, Muhammad Wahab Amjad

**Affiliations:** ^1^ Department of Pharmaceutical Chemistry, College of Pharmacy, Northern Border University, Rafha, Saudi Arabia; ^2^ Department of Pharmacy, Al-Yarmok University College, Diyala, Iraq; ^3^ Department of Pharmaceutical Chemistry, College of Pharmacy, National University of Science & Technology, Muscat, Oman; ^4^ Department of Chemistry, Faculty of Arts and Sciences, Northern Border University, Rafha, Saudi Arabia; ^5^ Medicinal Chemistry and Molecular Modelling Lab, Department of Pharmaceutical Chemistry, School of Pharmaceutical Education and Research, Jamia Hamdard, New Delhi, India; ^6^ Department of Pharmaceutics, College of Pharmacy, King Saud University, Riyadh, Saudi Arabia; ^7^ Irma Lerma Rangel College of Pharmacy, Texas A&M Health Science Center, College Station, TX, United States; ^8^ Center for Ultrasound Molecular Imaging and Therapeutics School of Medicine, University of Pittsburgh, Pittsburgh, PA, United States

**Keywords:** drug discovery, API (active pharmaceutical ingredient), pharmaceutical development, physicochemical interaction, patent

In general, drug products consumed by patients consist of two main constituents: the active pharmaceutical ingredient (a substance with a desired pharmacological activity) and the excipient (inert substances used to improve the delivery of the drug). Active pharmaceutical ingredient could be a salt or base or an ester form and these forms have different physicochemical and pharmacokinetic properties. For a drug to be formulated in a specific dosage form, it is necessary to prepare a suitable active pharmaceutical ingredient to comply with the dosage form characteristics. Thus, this Research Topic has been targeted by chemists and scientists interested in drug synthesis and development and focused mainly on active pharmaceutical ingredients (synthesis, physicochemical properties, chemical interactions, stability studies, and quality parameters).

Anti-ulcerative colitis effects of chemically characterized extracts from *Calliandra haematocephala* in acetic acid-induced ulcerative colitis by Rehman et al., explored the anti-inflammatory and the antioxidant effects of *C. haematocephala* extracts in a rat model of acetic acid-induced ulcerative colitis. Phytochemical analysis of methanolic and n-hexane extracts revealed the presence of many compounds like polyphenols, flavonoids, tannins, alkaloids, and sterols. Both extracts reduced colon ulceration and inflammation at all tested doses as revealed by the reduction in the macroscopic ulcer score and ulcer index. Mechanistically, the extracts upregulated the expression of the antioxidant enzyme superoxide dismutase and mitigated the expression of the proinflammatory mediators TNF-α and cyclooxygenase-2 (COX-2). Accordingly, the authors concluded that the anti-ulcerative colitis effect of *C. haematocephala* extracts is attributed to the antioxidant and anti-inflammatory effects, suggesting that *C. haematocephala* extracts could be a promising therapeutic approach for the development of therapeutic modalities to fight against ulcerative colitis.

Quality evaluation of compounds in leaves of six *Taxus* species based on ultra-high-performance liquid chromatography coupled with triple quadrupole mass spectrometry (UPLC-MS/MS) technique and chemometrics by (Cai et al.). The authors determined the chemical constituents in the leaves of six *Taxus* species by UPLC-MS/MS combined with chemometrics. The genus *Taxus* is widely distributed worldwide with 24 species and up to 400 toxoids. *Taxus* species are rich resources of medicinal agents like toxoids and flavonoids and are commonly used in traditional Chinese medicine (TCM). *Taxus* species cannot be distinguished based on the appearance of their leaves because they have similar morphological characteristics. Therefore, the authors employed UPLC-MS/MS, an advanced separation and analytical technique to identify the chemical constituents in the leaves of six *Taxus* species. Twenty-four components, including eight taxoids, four flavonols, five flavonols, two dihydroflavones, and five biflavones, were identified in the leaves of six *Taxus* species and screened by chemometric methods thereafter. Chemometric analysis identified six analytes as a reference to discriminate between different *Taxus* species.

Shah et al., carried out chemical finger printing and biological investigation of the isolated essential oil from the leaves of *Eucalyptus globulus* (Tasmanian blue gum). They characterized several low molecular weight compounds in the isolated essential oil using GC-MS. The oil exhibited promising antibacterial activity against both Gram-positive and Gram-negative pathogenic bacteria besides potent *in vitro* antioxidant activity. This study highlighted that the *E. globulus* essential oil could be a promising source of active pharmaceutical ingredients that may play an important role in combating various illnesses including bacterial and chronic diseases.

*Camellia fascicularis* leaves were used by the Peng et al., to identify bioactive compounds and to investigate antioxidant and antitumor activities of plant polar extracts. It is worth noting that the levels of active substances varied substantially in the leaves extracts prepared *via* sequential extraction method. Water extract of *C. fascicularis* remarkably inhibited the viability of HCCLM6 and HGC27 cells. via promoting the early apoptosis. This work provides a reference for the utilization of *C. fascicularis* as a natural medicine primarily as a source of antitumor agents in the aqueous extract and therefore merits further investigation.

